# Leptolide Improves Insulin Resistance in Diet-Induced Obese Mice

**DOI:** 10.3390/md15090289

**Published:** 2017-09-15

**Authors:** Pablo Villa-Pérez, Mercedes Cueto, Ana R. Díaz-Marrero, Carmen D. Lobatón, Alfredo Moreno, Germán Perdomo, Irene Cózar-Castellano

**Affiliations:** 1 Instituto de Biología y Genética Molecular, University of Valladolid-CSIC, Valladolid 47005, Spain; villaperezpablo@gmail.com (P.V.); clobaton@ibgm.uva.es (C.D.L.); amoreno@ibgm.uva.es (A.M.); 2 Instituto de Productos Naturales y Agrobiología (CSIC), La Laguna 38206, Spain; mcueto@ipna.csic.es; 3 Instituto Universitario de Bioorgánica “A. González”, University of La Laguna, La Laguna 38206, Spain; adiazmar@ull.edu.es; 4 School of Nursery, University of Burgos, Burgos 09001, Spain; gmperdomo@ubu.es

**Keywords:** leptolide, insulin resistance, obesity, type 2 diabetes, HepG2 cells

## Abstract

Type 2 diabetes (T2DM) is a complex disease linked to pancreatic beta-cell failure and insulin resistance. Current antidiabetic treatment regimens for T2DM include insulin sensitizers and insulin secretagogues. We have previously demonstrated that leptolide, a member of the furanocembranolides family, promotes pancreatic beta-cell proliferation in mice. Considering the beneficial effects of leptolide in diabetic mice, in this study, we aimed to address the capability of leptolide to improve insulin resistance associated with the pathology of obesity. To this end, we tested the hypothesis that leptolide should protect against fatty acid-induced insulin resistance in hepatocytes. In a time-dependent manner, leptolide (0.1 µM) augmented insulin-stimulated phosphorylation of protein kinase B (PKB) by two-fold above vehicle-treated HepG2 cells. In addition, leptolide (0.1 µM) counteracted palmitate-induced insulin resistance by augmenting by four-fold insulin-stimulated phosphorylation of PKB in HepG2 cells. In vivo, acute intraperitoneal administration of leptolide (0.1 mg/kg and 1 mg/kg) improved glucose tolerance and insulin sensitivity in lean mice. Likewise, prolonged leptolide treatment (0.1 mg/kg) in diet-induced obese mice improved insulin sensitivity. These effects were paralleled with an ~50% increased of insulin-stimulated phosphorylation of PKB in liver and skeletal muscle and reduced circulating pro-inflammatory cytokines in obese mice. We concluded that leptolide significantly improves insulin sensitivity in vitro and in obese mice, suggesting that leptolide may be another potential treatment for T2DM.

## 1. Introduction

Insulin resistance is one of the hallmarks of type 2 diabetes (T2DM) and obesity. Improvement of insulin sensitivity is an indispensable step to alleviate T2DM. The first phase of T2DM is characterized by pancreatic beta-cell compensation, displaying hyperinsulinemia in response to insulin resistance. Beta-cell overworking frequently end in dysfunction and cell death. At this point, decreased blood insulin levels exacerbate the onset of T2DM due to both insulin deficiency and resistance [1].

To this day, pharmacological management of T2DM patients aims to achieve the best possible glycemic control, while avoiding hypoglycemia. However, the natural history of T2DM includes multiple dysfunctions affecting the α-cells, β-cells, liver, skeletal muscle, adipose tissue, the gastrointestinal tract, kidney and brain, what has been termed the ominous octet [2]. This complex scenario of the pathophysiology of T2DM requires a shift in the current paradigm for the treatment of the disease.

A keystone in the management of diabetes is nutritional therapy along with regular physical activity. Considering that most people with T2DM are overweight or obese, weight loss is recommended to improve glycemic control [3]. In addition, regular physical activity has demonstrated significant health benefits [4]. When lifestyle interventions fail in the appropriate control of glucose homeostasis, T2DM is initially treated by monotherapy with oral agents, and eventually, it may require the combination of multiple drugs. In this way, insulin sensitizers and insulin secretagogues are safe therapies for T2DM treatment. Among the insulin sensitizers, metformin is successfully used as a first line pharmacotherapy for the treatment of T2DM patients. The major effect of this drug is the acute inhibition of hepatic gluconeogenesis through mechanisms involving the direct inhibition of the mitochondrial respiratory-chain complex I, leading to activation of AMP-activated protein kinase (AMPK) [5]. The pleiotropic effects of metformin lead to lower fasting blood glucose and insulin levels with minimal risk of hypoglycemia [6]. 

When glycemic control cannot be achieved using metformin as monotherapy, a second line of drugs is added to metformin treatment. Among these treatments, sulfonylureas improve insulin secretion by the regulation of the ATP-sensitive potassium channels in the plasma membrane of pancreatic β-cells [7]. Thiazolidinediones, agonists of nuclear peroxisome proliferator-activated receptor gamma (PPARγ), increase insulin sensitivity in liver and skeletal muscle [8]. Glinides stimulate insulin secretion rapidly and for a short period when needed [9]. Incretins, such as glucagon-like peptide 1 (GLP-1) receptor agonists, gastric inhibitory polypeptide/glucose-dependent insulinotropic peptide (GIP) and dipeptidyl-peptidase-4 (DPP-4) inhibitors, increase insulin secretion and regulate glucose homeostasis [10]. As the disease progresses, the β-cell function declines, and insulin therapy become necessary. The side effects of hypoglycemia and weight gain limit the efficacy of this therapy [11]. However, novel insulin preparations and delivery systems are available to optimize insulin therapy [12]. Regarding new glucose-lowering therapies, sodium glucose cotransporter 2 (SGLT2) receptor inhibitors lower blood glucose levels in an insulin-independent manner by increasing renal glucose excretion [13]. Promising new pharmacological targets are currently under evaluation, such as sirtuin agonists, which enhance insulin secretion and/or insulin sensitivity; or the inhibitors of protein tyrosine phosphatase 1B, which prolong the action of insulin [10].

Furanocembranolides are polyoxygenated diterpenoids, isolated from corals, in which a furanic ring and a β-lactone subunit are inserted in a cembrane skeleton [14]. Leptolide is a member of the furanocembranolide family, which was isolated for the first time by Gutiérrez et al. in 2005 [14] from the octocorals *Leptogorgia alba* and *Leptogorgia rigida* on the Pacific coast of Panama. It has been proposed that members of this family, such as pukalide, may function in nature as a defensive toxin against potential octocoral predators [15,16]. The potential pharmacological use of this family of compounds is largely unexplored. Although, few examples have been reported. Among them, lophotoxin is a neuromuscular toxin that binds selectively and irreversibly within the acetylcholine-recognition site of nicotinic acetylcholine receptors, thereby preventing acetylcholine from activating its receptor [17,18]. The antiproliferative and cytotoxic activities of some of these compounds have been studied against the cell lines L-929, K-562, HeLA, MDA-MB-231, A-549, HT-29 and P388 showing weak antiproliferative and cytotoxic properties [19,20,21]. Furthermore, the antiplasmodial activity of six furanocembranolides and the irregular pseudopterolide isolated from specimens of *Leptogorgia alba* and *L. rigida* was evaluated, and among them, leptolide and pukalide showed no biological activity against the parasite [14].

Leptolide, among other members of its family, has already been shown to increase pancreatic beta-cell proliferation in vitro, in INS1cells (Insulin secreting beta cell derived line) and primary cultures of rodent pancreatic islets [22]. In addition, epoxypukalide, another molecule of this family, has been shown to improve beta-cell protection in vitro and in vivo, in rodent islets and in a STZ (streptozotocin)-induced model of diabetes, respectively [22,23]. Interestingly, epoxypukalide also alleviates glucose intolerance in a preclinical model of type 1 diabetes [23]. Thus, furanocembranolides appear to be attractive molecules to maintain functional beta-cell mass and glycemic control.

In this work, we have extended our initial findings and explored the capability of leptolide to improve insulin sensitivity. To this end, we have assessed the capacity of leptolide to enhance insulin signaling in insulin-resistant hepatocytes and in the liver and skeletal muscle of diet-induced obese mice.

## 2. Materials and Methods

Leptolide purification, characterization and molecular structure were described previously [14]. Briefly, crude extracts from octocorals were subjected to fractionation. Leptolide was initially isolated as a novel compound with antiplasmodial activity, and its structure was determined by NMR and confirmed by single-crystal X-ray crystallography. 

### 2.1. Cell Culture

HepG2 cells were obtained from the American Type Culture Collection (ATCC, Manassas, VA, USA; #HB-8065). The cell line was originally isolated from a liver hepatocellular carcinoma of a 15-year-old Caucasian male. Cells were growth in DMEM (1X) supplemented with 4.5 g/L d-glucose, 0.6 g/L l-glutamine, 0.1 g/L sodium pyruvate and 10% fetal bovine serum. 

In order to analyze the effects of leptolide on the intracellular insulin signaling pathway, HepG2 cells were treated with 0.1 µM leptolide or vehicle (DMSO) during 24 h in medium without serum. Afterwards, 100 nM human insulin (Sigma, St. Louis, MO, USA) was added, and HepG2 cells were collected after 0, 5, 10, 15 and 30 min. To analyze the effects of leptolide in the setting of resistance, HepG2 cells were treated with 0.2 mM palmitate and 0.1 µM leptolide in serum-free medium for 24 h. Afterwards, 100 nM human insulin (Sigma, St. Louis, MO, USA) was added, and 15 min later, HepG2 cells were collected.

### 2.2. Animal Procedures

C57Bl6J male mice were purchase from Charles River Laboratory (Écully, France). Male mice were chosen for metabolic phenotyping to avoid the potential variability related to estrous cycle. Experimental procedures were approved by the Animal Care and Use Committee of the University of Valladolid (UVa), Valladolid, Spain, in accordance with the European and Spanish Guidelines for the Care and Use of Mammals in Research. Mice were fed with standard rodent chow and water ad libitum in ventilated cages in a 12:12-h light/dark cycle.

“Acute” administration of leptolide was performed in 12-week-old males fed a standard diet (SD) (33% protein; 58% carbohydrate; 9% fat) (#V1535, Ssniff, Soest, Germany) at the indicated doses (0.1 mg/kg and 1 mg/kg of body weight). “Chronic” administration of leptolide was performed in 6-week-old male mice fed a 60% kcal high fat diet (HFD) (20% protein; 20% carbohydrate; 60% fat) (#D12492, Research Diets, New Brunswick, NJ, USA) for 10 weeks. After 6 weeks of feeding with the HFD, mice were randomly divided into two groups, which were treated with once-daily ip injection of leptolide (0.1 mg/kg of body weight) or vehicle (DMSO) for another 4 weeks. All mice were maintained on HFD during the 4-week treatment. The day before sacrifice, mice were fasted overnight, followed by an ip insulin or saline injection, and 10 min later, mice were euthanized for liver and skeletal muscle tissues dissection as described previously [24].

Fasting or non-fasting blood was collected from the tail vein into capillary tubes precoated with potassium-EDTA (Sarstedt, Nümbrecht, Germany) for the preparation of plasma or the determination of blood glucose levels using the Breeze 2 glucometer (Bayer, Leverkusen, Germany) as previously described [24]. Insulin levels were measured using the ultrasensitive mouse ELISA assay (Mercodia, Uppsala, Sweden). Triglycerides were measured using a triglycerides kit (Biosystems, Barcelona, Spain). Cytokines and leptin were measured using Bio-Plex Luminex Immunoassays (Bio-Rad, Hercules, CA, USA). The detection limits of TNF-α, IL-1, IL6, insulin and leptin were 4.0 pg/mL, 1.6 pg/mL, 0.25 pg/mL, 0.025 pg/mL and 4.9 pg/mL respectively.

### 2.3. Glucose and Insulin Tolerance Tests, Glucose Decay and HOMA Indexes

The intraperitoneal glucose tolerance test (ip-GTT) was performed, 30 min after “acute” treatment or 4 weeks after “chronic” treatment, as previously described [24]. Briefly, mice were fasted overnight (15 h) following the recommended standard operating procedure for phenotyping mice by the Eumorphia Consortium [25]. Afterwards, mice were intraperitoneally injected with 2 g glucose/kg of body weight. Blood glucose levels were determined at 0, 15, 30, 60 and 120 min and plotted as a function of time. Likewise, the insulin tolerance test (ip-ITT) was performed, 30 min after “acute” treatment or 4 weeks after “chronic” treatment, as previously described [24]. For ip-ITT, non-fasted mice were injected (1 U/kg of body weight) with insulin (Lilly, Indianapolils, IN, USA). Blood glucose levels were determined at 0, 15, 30, 60 and 90 min and plotted as a function of time. Ip-GTT and ip-ITT experiments were performed using the same group of mice. The timeline of the experiments was first the ip-GTT assays. Then, we let mice recover for three days, and ip-ITT experiments were performed. Glucose decay was calculated from the glucose measurements obtained during the insulin tolerance test. The measurements were converted into natural logarithm (Ln); the slope was calculated using linear regression (time × Ln[glucose]) and multiplied by 100 to obtain the glucose decay constant rate per minute (%/min).

The homeostasis model assessment (HOMA) estimates steady state beta cell function (%B), insulin sensitivity (%S), the inverse of %S and the insulin resistance (HOMA-IR). The HOMA Calculator software is freely available at the University of Oxford, United Kingdom, at the web page www.dtu.ox.ac.uk/homacalculator.

### 2.4. Western-Blot Analysis

HepG2 cells were preincubated in the presence or absence of leptolide at the above indicated times and doses. At the end of the incubation period, culture media were discarded and cells collected and washed with ice-cold PBS, followed by homogenization in lysis buffer (20 mmol/L Tris·HCl, pH 7.5, 150 mmol/L NaCl, 1 mmol/L EDTA, 1 mmol/L EGTA, 1% (*vol*/*vol*) Triton X-100, 2.5 mmol/L sodium pyrophosphate, 1 mmol/L µ-glycerophosphate, 1 mmol/L Na_3_VO_4_, 1 µg/mL leupeptin and 1 mmol/L phenylmethylsulfonyl fluoride) plus protease inhibitors (Protease Inhibitor Cocktail; Sigma). After 10 min on ice, extracts were sonicated and centrifugated at 18,000× *g* for 10 min at 4 °C. Pellets were discarded, and solubilized proteins (~20–40 µg/sample) were resolved by 10% SDS-PAGE for anti-p-PKB (Ser473) (1:1000; Cell Signaling, Danvers, MA, USA) and electrotransferred onto polyvinylidene difluoride filters (PDVF Immobilon-P membrane (Millipore, Billerica, MA, USA)) for immunoblotting by conventional means. After probing with specific antibodies, the membranes were stripped and reprobed with antibody against actin (1:3000; Sigma) and PKB (1:1000; Cell Signaling). Signals were detected by chemiluminescence (Immun-Start Western Chemiluminescence Kit; Bio-Rad, Madrid, Spain), and band densitometry was quantified with Image J software (National Institutes of Health, Bethesda, MD, USA).

For animal tissues, liver and skeletal muscle from mice, stimulated or not with insulin, were homogenized with a polytron (OMNI, Kennesaw, GA, USA) in cell lysis buffer (Cell Signaling, USA) in the presence of protease/phosphatase inhibitors. As described for HepG2 cells, ~40–60 µg/sample were resolved in 10%-SDS PAGE for anti-pPKB, PKB and actin.

### 2.5. Statistical Analysis

Statistical analysis of data was performed using the GraphPad Prism Software 6.0 (La Jolla, CA, USA). Distributions were checked with the Kolmogorov–Smirnov test. Data are presented as the means ± S.E.M. Homogeneity of variance was performed using the Levene test. Comparisons between two groups were done using the unpaired Student’s *t*-test (if homogeneity of variance) or the Welch test (if heterogeneity of variance) when a variable was distributed normally; in the case of a non-parametric variable, the Mann–Whitney U-test was used. Comparisons between more than two groups were done using the one-way ANOVA or the Kruskal–Wallis test when a variable was distributed normally or non-normally, respectively. For post-hoc analyses, the Bonferroni test or Dunnett’s test was used if there was homogeneity or heterogeneity of variance, respectively. Differences were considered significant at *p* < 0.05.

## 3. Results

### 3.1. Leptolide Improves Insulin Signaling in HepG2 Cells 

**Figure 1:**
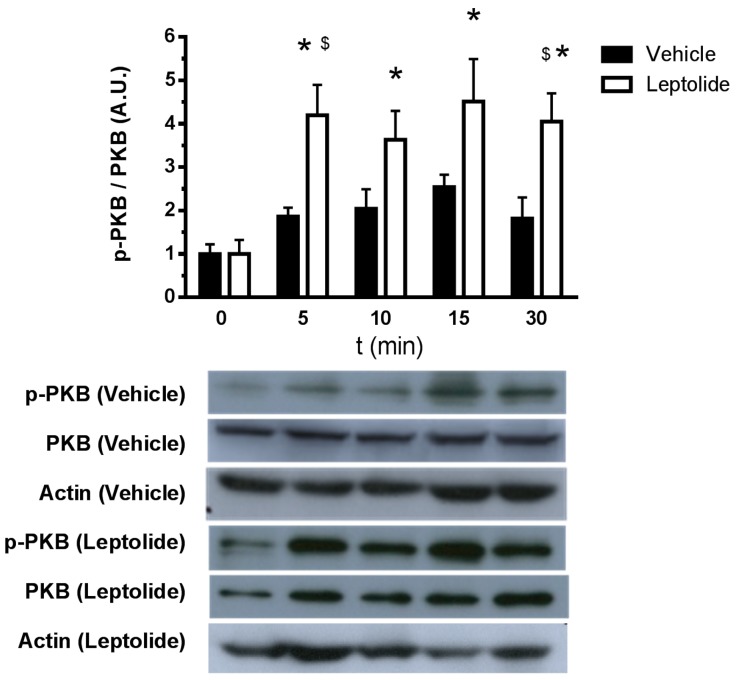
Leptolide augments insulin signaling in HepG2 cells. Western blot analysis of PKB and p-PKB in HepG2 cells treated with leptolide (0.1 µM) or vehicle and stimulated with 100 mM insulin for 5, 10, 15 and 30 min. Leptolide action on HepG2 cells enhanced insulin signaling at all time points. Values are the means ± S.E.M. of *n* = 6 per condition. The *y*-axis represents the ratio of phosphorylated versus total protein in arbitrary units. Actin was detected as a loading control. * *p* < 0.05 versus *t* = 0 by ANOVA; ^$^ *p* < 0.05 versus vehicle by ANOVA.

**Figure 2:**
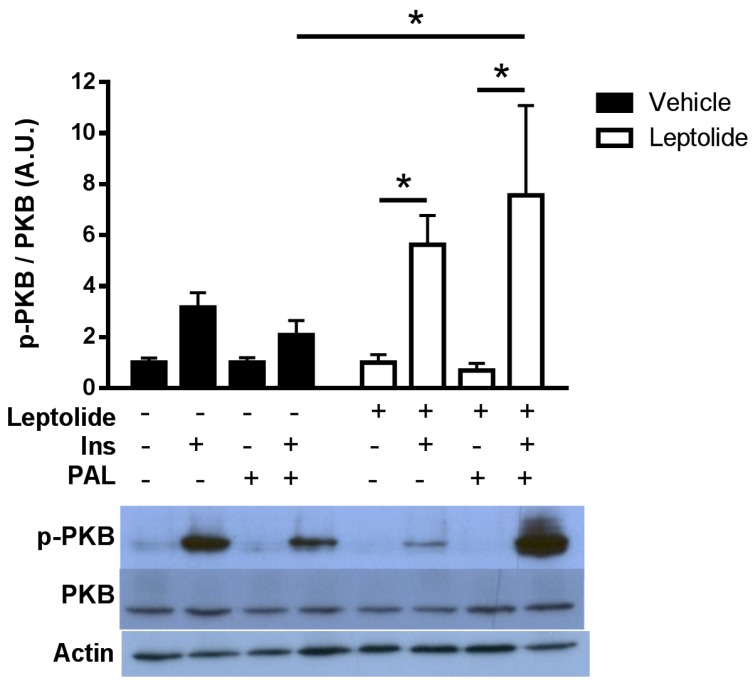
Leptolide prevents palmitate-induced insulin resistance in HepG2 cells. Western blot analysis of PKB and p-PKB in HepG2 cells treated with leptolide (0.1 µM) or vehicle, in the presence or absence of palmitate. Cells were stimulated with 100 mM insulin for 15 min. Leptolide action on HepG2 cells enhanced insulin signaling, preventing palmitate’s negative effect. Values are the means ± S.E.M. of n = 4 per condition. The *y*-axis represents the ratio of phosphorylated versus total protein in arbitrary units. Actin was detected as a loading control. * *p* < 0.05 versus non-stimulated insulin cells by ANOVA.

To study the effects of leptolide in basal and insulin resistance conditions, we have used a human hepatoma cell line (HepG2). To disregard the possibility of a cytotoxic effect of leptolide on HepG2 cells, 750,000 cells were plated and treated with vehicle or leptolide for 24 h (*n* = 4 independent experiments). Living cells were collected afterwards, and proteins were extracted and quantified showing no differences in protein content in leptolide- versus vehicle-treated cells (4.42 ± 0.53 μg/μL versus 4.30 ± 0.77 μg/μL). Vehicle-treated cells showed a time-dependent activation (5 min) of the insulin signaling pathway after insulin stimulation. Leptolide (0.1 µM) increased insulin signaling ~1.3–2.0-fold compared to vehicle-treated cells (Figure 1). This improvement in insulin sensitivity is sustained under palmitate-induced insulin resistance (Figure 2). HepG2 cells were treated with 0.2 mM palmitic acid for 24 h to cause insulin resistance, at the same time cells were treated with vehicle or 0.1 µM leptolide. Control cells showed an increased p-PKB/PKB ratio in basal conditions, which was impaired under insulin resistance conditions. Interestingly, leptolide treatment not only increases insulin signaling in basal conditions, but also counteracted palmitate-induced insulin resistance (Figure 2). These results highlight that leptolide is a molecule with the capacity of enhancing sensitivity at basal conditions and palmitate-induced insulin resistance in HepG2 cells.

### 3.2. Acute Treatment with Leptolide Improves Glucose Tolerance and Insulin Sensitivity in Lean Mice 

**Figure 3:**
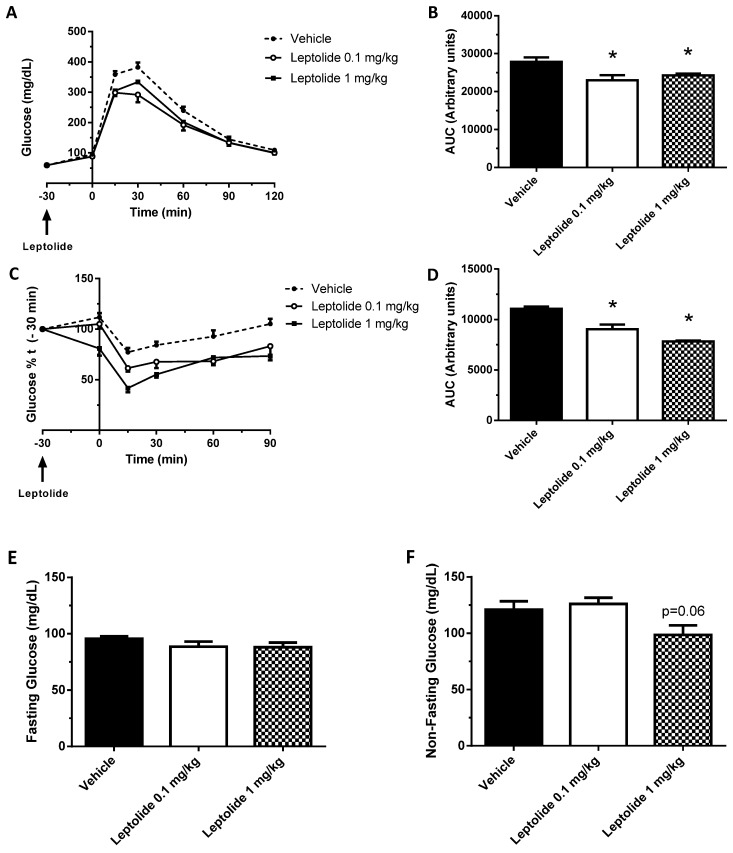
Acute leptolide administration improves glucose tolerance and insulin sensitivity in lean mice. C57Bl6J 12-week-old males fed a standard diet were injected with vehicle or leptolide 30 min before the intraperitoneal glucose tolerance test (ip-GTT) or the insulin tolerance test (ip-ITT). (**A**) Glucose tolerance test of mice injected with leptolide (0.1 mg/kg leptolide or 1 mg/kg) or vehicle. (**B**) Area under the curve of the ip-GTT. Glucose tolerance was improved using both leptolide concentrations compared to the vehicle. (**C**) Insulin tolerance test of mice injected with leptolide (0.1 mg/kg leptolide or 1 mg/kg) or vehicle. (**D**) Area under the curve of the ip-ITT. Insulin sensitivity was improved using both leptolide concentrations compared to vehicle. (**E**) Fasting and (**F**) non-fasting blood glucose levels after thirty minutes of leptolide administration. Fasting glucose levels were unchanged; meanwhile, only 1 mg/kg leptolide improved non-fasting glucose. Values are the means ± S.E.M. of *n* = 12 (vehicle); n = 6 (leptolide 0.1 mg/kg); n = 6 (leptolide 1 mg/kg). * *p* < 0.05 versus vehicle by ANOVA.

To corroborate our findings in vitro, we acutely injected leptolide at two different concentrations (0.1 and 1 mg/kg) in C57BL6J male mice. Each dose of leptolide and its respective control were administered as part of independent experiments, and they were performed on separate days. Vehicle data were pooled from both experiments. Leptolide was injected 30 min before ip-GTT in fasted mice (time point −30). At the same time, plasma glucose levels were assessed (time point −30). Afterwards, a bolus of glucose was administered intraperitoneally as described in the Materials and Methods Section. Plasma glucose levels were monitored at 15, 30, 60, 90 and 120 min after glucose challenge. As shown in Figure 3A,B, both leptolide concentrations displayed improved glucose tolerance. In a different group of experiments, leptolide was injected previous to ip-ITT showing increased insulin sensitivity at both leptolide concentrations (Figure 3C,D). Likewise, acute administration of leptolide (0.1 and 1 mg/kg) improved insulin sensitivity in lean mice (Figure 3C,D). Consistent with these effects of leptolide on whole-body glucose homeostasis, non-fasting plasma glucose levels were reduced (*p* = 0.06) in mice treated with a high dose of leptolide (Figure 3E,F). Collectively, these results demonstrate that both doses of leptolide have a positive effect on improving insulin sensitivity and glucose homeostasis. In view of these data, we have chosen the lowest dose of leptolide to perform the next experiments in diet-induced obese mice. 

### 3.3. Prolonged Leptolide Administration Improves Liver and Muscle Insulin Resistance in Diet-Induced Obese Mice

**Figure 4:**
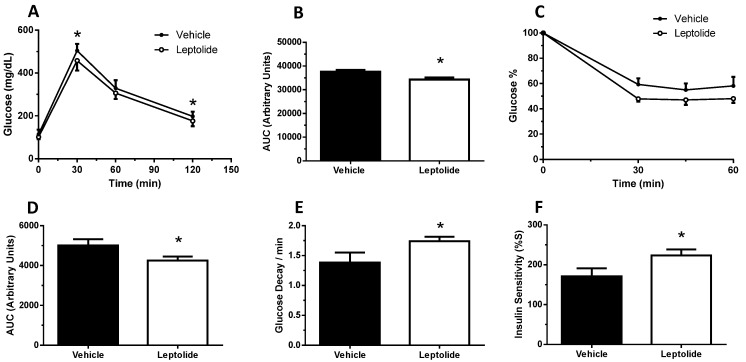

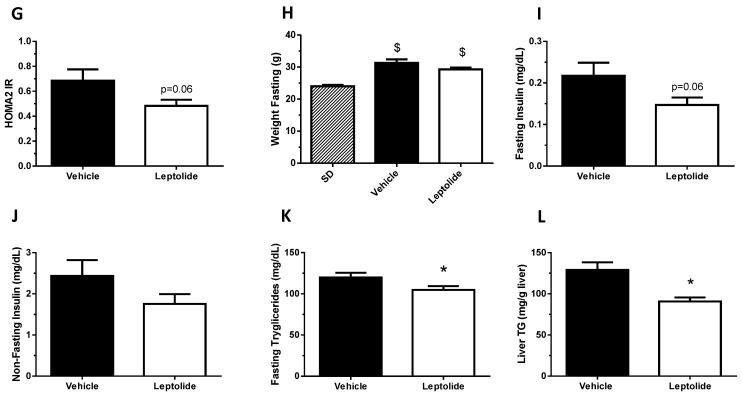
Prolonged leptolide administration improves insulin sensitivity in a preclinical model of insulin resistance. C57BL6J male mice were fed an HFD for ten weeks. The last four weeks, mice were injected intraperitoneally with leptolide or saline once a day. Afterwards, insulin sensitivity and plasma levels of insulin and triglycerides were assessed. (**A**) Glucose tolerance test of mice treated with vehicle or 0.1 mg/kg leptolide. (**B**) Area under the curve of the ip-GTT. Glucose tolerance improved in leptolide- compared to vehicle-treated mice. (**C**) Insulin tolerance test of mice treated with vehicle or 0.1 mg/kg leptolide. (**D**) Area under the curve of the ip-ITT. Insulin sensitivity improved in leptolide- compared to vehicle-treated mice. (**E**) Glucose decay after first 30 min of insulin injection during ip-ITT. (**F**) Insulin sensitivity index (%S). (**G**) HOMA index. Insulin sensitivity and HOMA indexes showed improved insulin sensitivity in HFD + leptolide mice. (**H**) Fasting body weight. (**I**) Fasting and (**J**) non-fasting plasma insulin levels. Insulin levels were non-significantly decreased. (**K**) Fasting plasma triglycerides levels were decreased in mice treated with leptolide. (**L**) Liver TG (triglyceride) content was decreased in parallel with plasma triglyceride levels. Values are the means ± S.E.M. of *n* = 12 per group. * *p* < 0.05 versus vehicle by Student’s *t*-test; ^$^ *p* < 0.05 versus SD by ANOVA.

**Figure 5:**
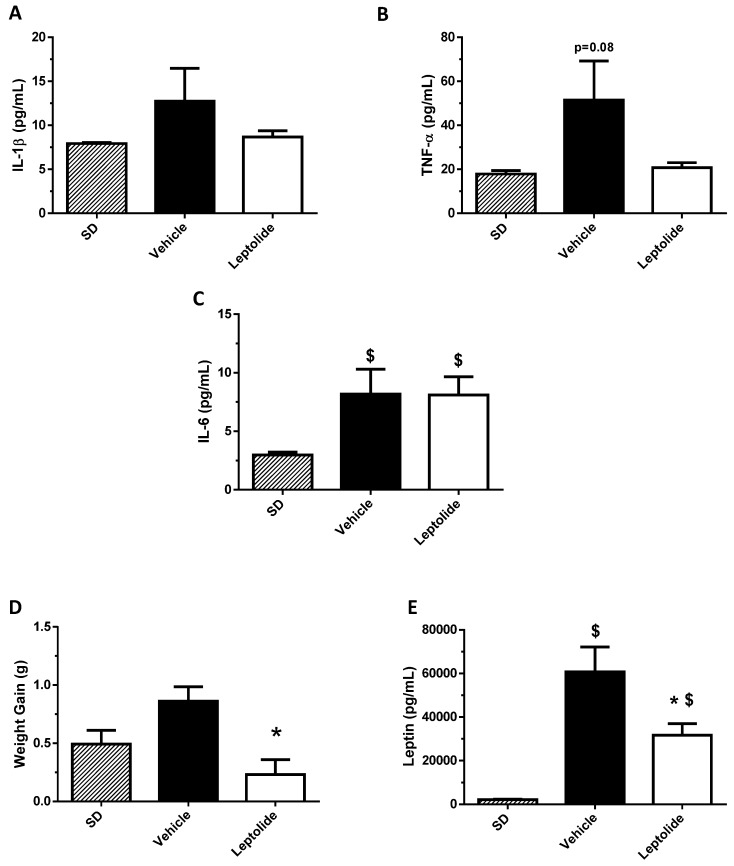
Prolonged leptolide administration decreases body weight gain and circulating leptin. C57BL6J mice were treated as described in Figure 4; standard diet (SD) were used as controls. Animals were bled before sacrifice, and cytokines were measured from their plasmas: (**A**) IL-1β; (**B**) TNF-α; (**C**) IL-6; (**D**) body weight gain; and (**E**) leptin. Weight gain, TNF-α and leptin plasma levels were decreased in leptolide- versus vehicle-treated mice. The detection limits of TNF-α, IL-1, IL6 and leptin were 4.0 pg/mL, 1.6 pg/mL, 0.25 pg/mL and 4.9 pg/mL, respectively.* *p* < 0.05 versus vehicle by ANOVA; ^$^ *p* < 0.05 versus SD by ANOVA.

**Figure 6:**
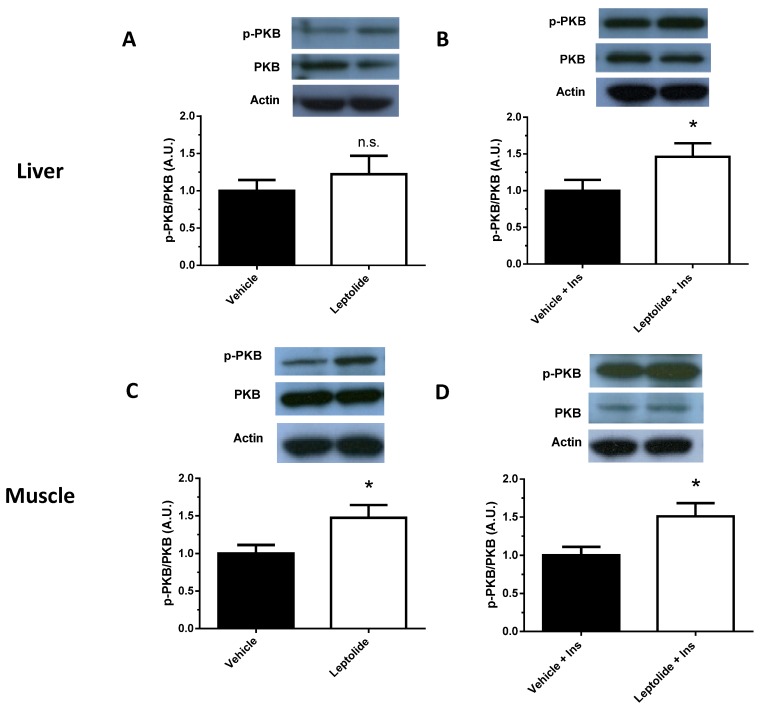
Prolonged leptolide administration improves peripheral insulin signaling in a preclinical model of insulin resistance. C57BL6J mice were treated as described in Figure 4. To analyze the intracellular insulin signaling pathway, mice were intraperitoneally injected with insulin or saline for 10 min. Afterwards, the insulin signaling pathway was analyzed in liver and skeletal muscle tissues by Western blotting. Western blot analysis of PKB and p-PKB in the liver of mice injected with (**A**) saline or (**B**) insulin. Insulin signaling was enhanced in livers of leptolide- versus vehicle-treated mice. Values are the means ± S.E.M. of *n* = 6 per group. * *p* < 0.05 versus vehicle by Student’s *t*-test. Western blot analysis of PKB and p-PKB in the skeletal muscle of mice injected with (**C**) saline or (**D**) insulin. Insulin signaling was enhanced in skeletal muscles of leptolide- versus vehicle-treated mice. Values are the means ± S.E.M. of *n* = 6 per group. Actin was detected as a loading control. * *p* < 0.05 versus vehicle by Student’s *t*-test.

Next, we tested the hypothesis that administration of leptolide improves insulin sensitivity in obese mice. To this end, C57BL6J mice were fed a high fat diet (60% kcal of fat) for ten weeks. The last four weeks, mice were intraperitoneally injected with leptolide (0.1 mg/kg) once a day. At the end of the treatment, insulin sensitivity was assessed by ip-ITT and the HOMA index in all mice. To investigate the impact of leptolide in the intracellular insulin signaling pathway in liver and skeletal muscle tissues, one-half of mice (control and leptolide groups) were injected with insulin for 10 min, whereas the other half was injected with saline. Afterwards, mice were euthanized and sacrificed for the dissection of liver or skeletal muscle tissues. Data in Figure 4, Figure 5 and Figure 6 were pooled from two discrete experiments using two different groups of mice.

Prolonged administration of leptolide improved glucose tolerance (Figure 4A,B) and insulin sensitivity (Figure 4C,D) in obese mice. Consistently, glucose decay during the ip-ITT during the first 30 min was improved in the leptolide-treated group (Figure 4E). Furthermore, the insulin sensitivity index (%S) was significantly increased (Figure 4F), which was paralleled with a reduced HOMA-IR index (*p* = 0.06; Figure 4G), although body weight was not significantly decreased (Figure 4H). In addition, fasting and non-fasting plasma insulin levels were reduced in leptolide-treated group mice (Figure 4I,J), which is consistent with the notion of improving insulin sensitivity. Finally, plasma triglyceride levels were significantly reduced in obese mice treated with leptolide (Figure 4K), which nicely correlates with a 30% reduction in liver triglyceride content in leptolide- versus vehicle-treated mice (Figure 4L). Taken together, these data demonstrate that chronic leptolide administration counteracted insulin resistance and improved lipid metabolism in diet-induced obese mice.

To gain insight into the molecular mechanisms by which leptolide improved insulin resistance, we analyzed levels of circulating pro-inflammatory cytokines. As shown in Figure 5, chronic leptolide treatment was associated with a non-statistically-significant decrease in blood levels of IL-1β and TNF-α, but IL-6 levels remained unchanged (Figure 5A–C). Likewise, leptolide significantly reduced the weight gain during the last week of the treatment (Figure 5D), in parallel with reduced plasma leptin levels in obese mice (Figure 5F). 

To further investigate the impact of prolonged administration of leptolide on obese mice, the activation of the intracellular insulin signaling pathway was assessed in the liver and skeletal muscle of obese mice. As shown in Figure 6, leptolide improved at basal and insulin-stimulated conditions, with the phosphorylation of PKB in liver and skeletal muscle tissue. These results are in good agreement with the effect of leptolide on insulin sensitivity in obese mice.

## 4. Discussion

T2DM is a metabolic disease characterized by insulin resistance, which may be joined with reduced insulin production and secretion. New drugs for T2DM treatment should include the capability of improving insulin sensitivity and protect functional beta-cell mass. We have demonstrated that furanocembranolides are promising molecules in the treatment of T2DM. Thus, we have shown that furanocembranolides induce beta-cell proliferation and protection, maintaining functional beta-cell mass and insulin production in type 1 diabetes [22,23]. In this work, we have shown that leptolide acts as an insulin sensitizer, improving insulin sensitivity and intracellular insulin signaling in the liver and muscle of obese mice. 

Obesity is associated with insulin resistance [26]. In this work, we have shown that leptolide reduced weight gain, which was parallel with lower pro-inflammatory circulating cytokines and leptin. These effects may mediate one of the molecular mechanisms by which leptolide improved insulin sensitivity in skeletal muscle and liver tissues. In this line of thinking, we have previously demonstrated that epoxypukalide, a member of the furanocembranolide family, protected primary rat β-cell cultures from a cocktail of pro-inflammatory cytokines including IL-1β, IFN-γ and TNF-α [22]. 

T2DM is a complex disease that hardly can be managed using mono-therapeutic approaches and often requires double or triple therapeutic combinations depending on the disease progression [1,27]. In addition, the comorbidities associated with T2DM, such as cardiovascular disease, require specific medication. Thus, polymedicated T2DM patients are more susceptible to lower adherence to treatment, increased risk of harmful drug interactions and increased healthcare spending [28]. In this line of argumentation, the discovery and development of a new class of drugs with pleiotropic effects are highly relevant for T2DM management. 

Leptolide is an exceptional drug that can enhance the post-receptor intracellular insulin signaling cascade and increases beta-cell proliferation [22]. These characteristics make leptolide an attractive molecule to explore the possibility of overcoming polymedication in T2DM patients. Further studies are warranted for the determination of the optimum drug dosage, timing of dosages, systemic bioavailability and the route of administration to enhance leptolide activity in vivo.

The functional activity of this family of natural compounds on mammalian cells is mostly unknown. Our data suggest that one potential mechanism of action of these compounds is through the activation of the insulin signaling pathway in multiple tissues. Upon binding of insulin, the kinase domains of the insulin receptor (IR) are activated by autophosphorylation on tyrosine residues, resulting in tyrosine phosphorylation of insulin receptor substrate (IRS) proteins [29]. In the liver, IRS2 is important for the integration of the insulin signal and the metabolic control of the hepatocytes [30]. Phosphorylated-IRS proteins allow the association and activation of phosphatidylinositol 3-kinase (PI3K), leading to the production of phosphatidylinositol-3,4,5-triphosphate (PIP3), a lipid second messenger located on the plasma membrane. PIP3 allows the recruitment and activation of 3-phosphoinositide-dependent protein kinase 1 (PDK1) and serine/threonine protein kinase AKT (also known as PKB) [29]. These proteins (IRS, PI3K and PKB) are considered three critical nodes of the canonical insulin receptor signal transduction network [31]. In the liver, AKT2 is one of the major mediators of the metabolic effects of insulin [32]. For this reason, we evaluated the phosphorylation levels of PKB in obese mice. 

Here, we show that leptolide enhances the phosphorylation of PKB in liver and skeletal muscle tissues of a preclinical model of insulin resistance. However, the insulin signaling pathway also regulates cell growth and differentiation, emanating from the IRS node. The regulation of these processes is mediated by the Raf/Ras/MEK/MAPK (mitogen-activated protein kinase, also known as ERK or extracellular signal regulated kinase) pathway [31]. Interestingly, we have previously demonstrated that epoxypukalide is able to induce the ERK1/2 pathway, but not PKB in pancreatic beta-cells [22].

Leptolide and epoxypukalide possess the same carbon skeleton and contain the same macrocycle. The only structural difference between them lies at C-18, which is oxidized to a methyl ester in epoxypukalide and to an aldehyde in leptolide [22,33]. Our results suggest that this difference is responsible for their ability to activate different pathways depending on cell type and cell environment. Slight variations in functional groups of furanocembranolides have been reported to make a difference in their activities [14,17]. The way these molecules enter the cell or activate signaling pathways is not known yet. Further research is necessary to address these open questions. 

In conclusion, our findings demonstrate the feasibility of furanocembranolides as a new therapeutic strategy to treat T2DM.
